# Supplementary material for: Neuron hemilineages provide the functional ground plan for the Drosophila ventral nervous system
Source: eLife. 2015 Jul 20;4:e04493. doi: 10.7554/eLife.04493 (PMC4525104; doi:10.7554/eLife.04493)
Supplement: Supplementary file 1. — Genotypes used for anatomical and behavioral characterization of the hemilineages and the amount of off-target expression in the ventral nervous system of each. DOI: http://dx.doi.org/10.7554/eLife.04493.039 [file elife04493s001.docx]

**Supplemental File 1.** Genotypes used for anatomical and behavioral characterization of the hemilineages and the amount of off-target expression in the ventral nervous system of each.

| **Hemilineage** | **Genotype** | **Off-target expression** | **Use** |
| --- | --- | --- | --- |
| 1A | R22G11-LexA, LexAop-GFP | 10-20 neurons in abdominal neuromeres | Anat |
|  | R22G11-LexA, LexAop-TRPA1 |  | Behav |
| 1B | R58F02-GAL4, nSyb-GAL80, UAS-[hPR]-flp, act>STOP>LexA, LexAop-GFP | Strong expression in hemilineage 6A neurons projecting to tectulum | Anat |
| 2B | R50G08-GAL4, UAS-IVS-GFP | Expression in abdominal homologs of 2B neurons | Anat |
|  | R50G08-GAL4, UAS-TRPA1 |  | Behav |
| 3A | R31H10-GAL4, nSyb-GAL80, UAS-flp, act>STOP>LexA, LexAop-GFP | Intense expression in sensory neurons | Anat |
| 3B | R23B05-LexA, LexAop-GFP | ~10 weak neurons in thorax | Anat |
|  | R23B05-LexA, LexAop-TRPA1 |  | Behav |
| 5B | R86D02-GAL4, nSyb-GAL80, UAS-flp, act>STOP>LexA, LexAop-GFP | 1-2 thoracic neurons/segment;  ~30 abdominal neurons | Anat |
|  | R86D02-GAL4, nSyb-GAL80, UAS-flp, nSyb-LexA, LexAop>STOP>TRPA1 |  | Behav |
| 6A | R35A03-GAL4, nSyb-GAL80, UAS-flp, act>STOP>LexA, LexAop-GFP | ~ 5 weak cells/thoracic segment; ~30 abdominal neurons | Anat |
|  | R35A03-GAL4, nSyb-GAL80, UAS-flp, nSyb-LexA, LexAop>STOP>TRPA1 |  | Behav |
| 6B | R46C11-GAL4, nSyb-GAL80, UAS-flp, act>STOP>LexA, LexAop-GFP | Weak expression in 1-2 neurons/ thoracic segment | Anat |
|  | R46C11-GAL4, nSyb-GAL80, UAS-flp, nSyb-LexA, LexAop>STOP>TRPA1 |  | Behav |
| 7B | R65A12-GAL4, nSyb-GAL80, UAS-flp, act>STOP>LexA, LexAop-GFP | <5 weak expressing neurons in abdomen, outside of 7B cluster in A1 | Anat |
|  | R65A12-GAL4, nSyb_GAL80, UAS-flp, nSyb-LexA, LexAop>STOP>TRPA1 |  | Behav |
| 8A | R69H11-GAL4, UAS-GFP | ~2-4 neurons per abdominal neuromere | Anat |
|  | R69H11-GAL4, UAS-TRPA1 |  | Behav |
| 8B | R09D08-GAL4, nSyb-GAL80, UAS-[hPR]-flp, act>STOP>LexA, LexAop-GFP | Limited expression in another hemilineage | Anat |
| 9A | R52E12-GAL4, UAS-GFP | none | Anat |
|  | R52E12-GAL4, UAS-TRPA1 |  | Behav |
| 10B | R13B08-GAL4, nSyb-GAL80, UAS-[hPR]flp, act>STOP>LexA, LexAop-GFP | ~2-4 neurons per abdominal neuromere | Anat |
|  | R13B08-GAL4, nSyb-GAL80, UAS-[hPR]flp, nSyb-LexA, LexAop>STOP>TRPA1 |  | Behav |
| 11A/B | R26B05-GAL4, nSyb-GAL80, UAS-[hPR]-flp, act>STOP>LexA, LexAop-GFP | 2-3 weak neurons/segment in thorax; small, segmental sensory projections in abdomen | Anat |
|  | R26B05-GAL4, nSyb-GAL80, UAS-[hPR]-flp, nSyb-LexA, LexAop>STOP>TRPA1 |  | Behav |
| 12A | R24B02-GAL4, nSyb-GAL80, UAS-[hPR]-flp, act>STOP>LexA, LexAop-GFP | 1-2 neurons per abdominal neuromere | Anat |
|  | R24B02-GAL4, nSyb-GAL80, UAS-[hPR]-flp, LexAop>STOP>TRPA1, nSyb-LexA |  | Behav |
| 12B | R15D11-GAL4, nSyb-GAL80, UAS-[hPR]-flp, act>STOP>LexA, LexAop-GFP | none | Anat |
|  | R15D11-GAL4, nSyb-GAL80, UAS-[hPR]-flp, LexAop>STOP>TRPA1, nSyb-LexA |  | Behav |
| 13A | R49C05-GAL4, nSyb-GAL80, UAS-flp, act>STOP>LexA, LexAop-GFP | Stocastic expression in other lineages, especially 23B | Anat |
| 13B | R41G09-GAL4, UAS-IVS-GFP | Weak expression in about 10 neurons per thoracic neuromere | Anat |
|  | R41G09-GAL4, UAS-TRPA1 |  | Behav |
| 14A | R80G10-GAL4, nSyb-GAL80, UAS-hPR-flp, act>STOP>LexA, LexAop-GFP | Stocastic expression in hemilineage 2 neurons | Anat |
| 15B | R12F11-GAL4, nSyb-GAL80, UAS-flp, act>STOP>LexA, LexAop-GFP | Contaminated with dorsal hemilineages | Anat |
| 18B | R27A09-GAL4, nSyb-GAL80, UAS-flp, act>STOP>LexA, LexAop-GFP | Abdominal motorneurons | Anat |
|  | R27A09-GAL4, nSyb-GAL80, UAS-flp, nSyb-LexA, LexAop>STOP>TRPA1 |  | Behav |
| 19A | R32E04-GAL4, UAS-GFP | 1-2 neurons per thoracic neuromere; ~ 10-20 neurons in abdomen | Anat |
|  | R32E04-GAL4, UAS-TRPA1 |  | Behav |
| 19B | R84E06-GAL4, nSyb-GAL80, UAS-flp, act>STOP>LexA, LexAop-GFP | Strong expression in haltere afferents | Anat |
| 20A,22A | R24G06-LexA, LexAop-GFP | none | Anat |
|  | R24G06-LexA, LexAop-TRPA1 |  | Behav |
| 21A | R26H05-GAL4^AD^, R51H05-GAL4^DBD^, UAS-flp, act>STOP>LexA, LexAop-GFP | 3-5 neurons/ thoracic segment; abdominal motoneurons | Anat |
|  | R26H05-GAL4^AD^, R51H05-GAL4^DBD^, UAS-flp, LexAop>STOP>TRPA1, nSyb-LexA |  | Behav |
| 23B | R77C10-GAL4, UAS-GFP | none | Anat |
|  | R77C10-GAL4, UAS_TRPA1 |  | Behav |
| 24B | R15A03-GAL4, nSyb-GAL80, UAS-flp, nSyb-LexA, LexAop>STOP>GFP | Occasional descending axons; <10 abdominal neurons | Anat |
|  | R15A03-GAL4, nSyb-GAL80, UAS-flp, nSyb-LexA, LexAop>STOP>GFP |  | Behav |
